# Integrating Finite Element Death Technique and Bone Remodeling Theory to Predict Screw Loosening Affected by Radiation Treatment after Mandibular Reconstruction Surgery

**DOI:** 10.3390/diagnostics10100844

**Published:** 2020-10-19

**Authors:** Le-Jung Wu, Kai-Hung Hsieh, Chun-Li Lin

**Affiliations:** 1 Division of Radiation Therapy, Far Eastern Memorial Hospital, New Taipei City 220, Taiwan; mvphoton@gmail.com; 2 Department of Biomedical Engineering, National Yang-Ming University, Taipei 112, Taiwan; etony83719@gmail.com

**Keywords:** finite element, element death, radiation treatment, 3D printing, screw loosening

## Abstract

This study developed a numerical simulation to understand bone mechanical behavior and micro-crack propagation around a fixation screw with severe mandibular defects. A mandible finite element (FE) model was constructed in a rabbit with a right unilateral body defect. The reconstruction implant was designed to be fixed using six screws distributed on the distal and mesial sides. The element death technique provided in FE analysis was combined with bone remodeling theory to simulate bone necrosis around the fixation screw in which the strain value reached the overload threshold. A total of 20 iterations were performed to observe the micro-crack propagation pattern for each screw according to the high strain locations occurring in each result from consecutive iterations. A parallel in vivo animal study was performed to validate the FE simulation by placing specific metal 3D printing reconstruction implants in rabbits to compare the differences in bone remodeling caused by radiation treatment after surgery. The results showed that strain values of the surrounding distal bone fixation screws were much larger than those at the mesial side. With the increase in the number of iteration analyses, the micro-crack prorogation trend for the distal fixation screws can be represented by the number and element death locations during the iteration analysis process. The corresponding micro-movement began to increase gradually and induced screw loosening after iteration calculation. The strained bone results showed that relatively high bone loss (damage) existed around the distal fixation screws under radiation treatment. This study concluded that the FE simulation developed in this study can provide a better predictive diagnosis method for understanding fixation screw loosening and advanced implant development before surgery.

## 1. Introduction

The fibula free flap is the gold standard surgery for the vascularized graft used to reconstruct severe defects in the mandible because of its versatility, predictability, and favorable fibula bone quantity for dental implants to facilitate prosthetic rehabilitation [1,2,3,4]. The reconstruction plate and fixation screws were used to secure the bone graft in the lower mandible border to bridge the mandibular stumps to bear the occlusal load to maintain fixation stability during the bone healing phase [1,5,6,7,8]. However, hidden worries and the risk of fixation screw loosening still exist after surgery.

Fixation screw loosening is caused by insufficient retention between the screw and bone. The reason may be related to the radiation treatment performed after surgery. The blood vessels and cells in the bone cannot transfer nutrients normally and lose the ability to self-repair osteoradionecrosis, which directly reduces the bone retention capability due to hypoxia caused by radiation treatment (hypoxic), decreased cell activity (hypocellular), and vascular atrophy (hypovascular), i.e., the 3H theory proposed by Marx et al. [9,10,11].

The mechanostat theory indicated that bone remodeling is related to the mechanical load. Bone loss may occur when the mechanical load falls below a lower threshold or above an upper threshold (bone overloading) [12,13,14,15,16]. The upper threshold range might be decreased due to the influence of 3H theory. This means that bone withstanding an excessive mechanical load becomes worse and damage is more likely to occur under certain load conditions. Bone screw loosening in mandibular reconstruction is a biomechanical complication. Enhanced understanding of the formative bone response to loads (modeling) and maintenance for an integrated state (remodeling) could improve the clinical treatment. Bone resorption surrounding the fixation screw is a major problem as it causes looseness at the bone–screw interface, thus undermining the implant system integrity [1,8,17]. When radiotherapy is performed after the operation, the bone surrounding the fixation screw is more likely to be damaged when receiving a low mechanical load due to the bone remodeling ability becoming out of balance.

Quantification stress, strain, or strain energy density has been proposed as the mechanical stimulus for bone remodeling [13,14,15,16,17]. Mathematical algorithms with a bone overload mechanical threshold can be combined with the element birth and death technique provided in advanced finite element (FE) analysis. This approach can be employed to put forward quantitative numerical models for bone damage prediction [13,14,15,16,17]. A better understanding of the damage process initiation and accumulation around the fixation screw could provide information for screw loosening prediction and improved implant reconstruction design. 

This study combined the finite element death technique and bone remodeling theory to investigate the mechanical behavior and crack accumulation around a fixation screw in a rabbit with severe mandibular defects after surgery with radiation treatment. A parallel in vivo animal experiment that placed a titanium 3D printing reconstruction implant and screws into a specific rabbit with a mandibular defect and radiation treatment performed after surgery was carried out for validation.

## 2. Materials and Methods

### 2.1. Finite Element Model Generation

**Figure 1:**
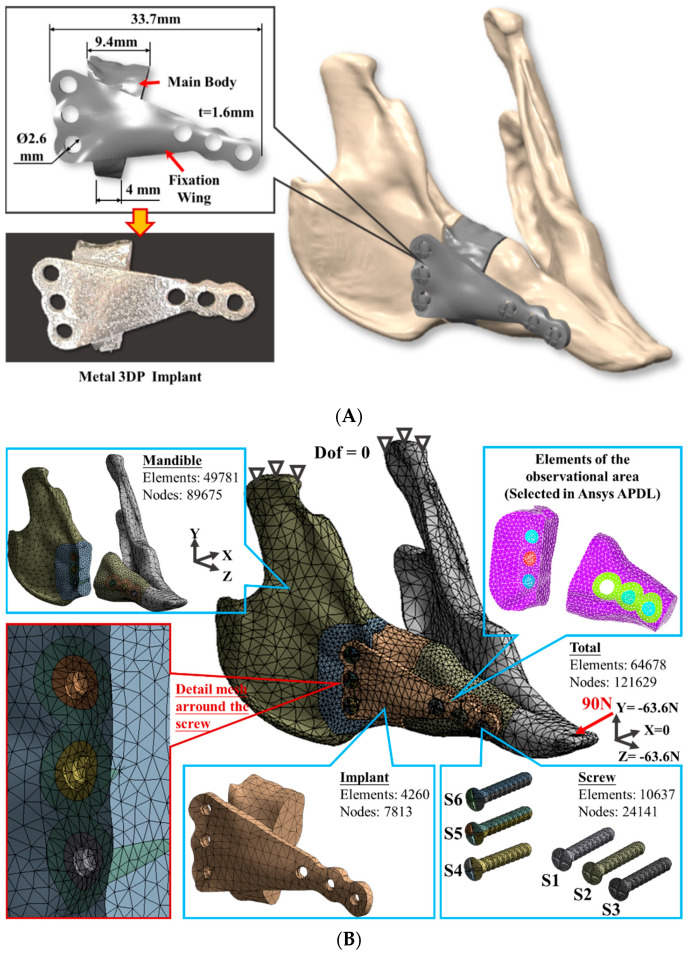
(**A**) Solid model of the rabbit mandible with unilateral defect at the right posterior teeth area and placed with a specific reconstruction implant; (**B**) FE (finite element) mesh model of simulated mandible with reconstruction implant and fixation screw (S4, S5, and S6 at distal side and S1, S2, and S3 at mesial side) and detailed mesh pattern around the fixation screws.

A digital mandible solid model of a New Zealand rabbit was constructed by stacking cross-section image contours of various hard tissues (cortical and cancellous bone) that were obtained from a series of computed tomography (CT, LightSpeed Plus, GE Medical System, WIS, Chicago, IL, USA) images with 1 mm interval. A unilateral body defect region was defined at the right posterior tooth area to perform the reconstruction procedure. The reconstruction implant was designed to include the main body for appearance consideration and a fixation wing for retention/ensuring the primary stability requirement with adjacent bones. The total length of the implant was 33.7 mm, with wing thickness of 1.6 mm (Figure 1A). The screw hole size was 2.6 mm in diameter. Three holes were distributed in a vertical line (S4, S5, and S6) on the distal side and three other holes in a horizontal line (S1, S2, and S3) on the mesial side, all designed to insert and fully connect the implant to the bone surfaces (Figure 1). 

The corresponding fixation screws with thread details were generated in a CAD system (Creo Parametric v2.0, PTC, Needham, MA, USA) and assembled with the remaining mandibular bone and reconstruction implant (including the main body and the fixation wing) in the ANSYS Workbench (ANSYS Workbench v18.2, ANSYS Inc., Pittsburgh, PA, USA) for further simulation. A mesh convergence test was performed to control the strain energy and displacement variations smaller than 5% for models with different element sizes. The FE model was then generated using quadratic ten-node tetrahedral structural solid elements (Figure 1B). 

In order to perform the element death technique, the mesh sizes around the fixation screws were further arranged into 3 layers. The size of each layer was gradually increased from the screw axis to the radial and the mesh sizes were set to 0.6 mm, 0.8 mm, and 1 mm in order. The element and node number were 49,781 and 89,675 for the mandibular bone, 4260 and 7813 for the reconstruction implant, and 10,637 and 24,141 for the fixation screw. A total of 64,678 elements and 121,629 nodes were accommodated in the simulated model. Cortical, cancellous bones and reconstruction implant/screws were assumed with linear elastic and isotropic properties. Elastic modulus and Poisson’s ratio material property values were adopted from the relevant literature [1] (Table 1). Nodes on the condyle were constrained in all directions to prevent movement as the boundary conditions and a concentrated oblique occlusal load with 90N was applied to the incisor as the load condition (Figure 1B) [18,19].

### 2.2. Micro-Crack Propagation Simulation Using Element Death Analysis

This study combined bone remodeling theory and finite element death technology to simulate bone necrosis behavior around the fixation screw from radiation treatment after surgery [1,13,14,15,16,17,18,19,20,21]. The accumulation of osteonecrosis in the elements around the fixation screw at different time points can be simulated using different iterative simulation times (denoted as IT_X; X was the number of iterations) [22]. Observing the bone necrosis accumulation at different time points can roughly identify the micro-crack propagation trend.

**Figure 2:**
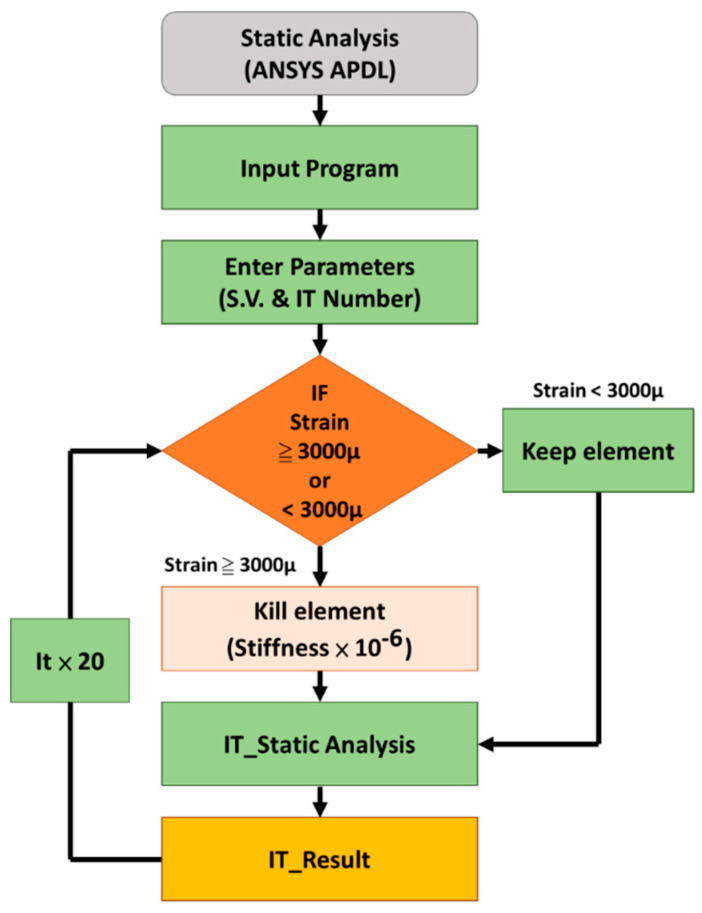
Element death iterative calculation in ANSYS. IT: iteration of calculation.

An in-house APDL (ANSYS parametric design language) iterative code with the element death technique was developed to simulate the micro-crack propagation around the fixation screw. Figure 2 shows a flowchart of one iterative calculation. This code automatically judged and selected the elements around the fixation screws (S1 to S6) so that the strain value exceeded 3000 μ because this value was set as the overload threshold for bone remodeling after radiotherapy [13,14,15,16,17,18,19,23,24]. The selected elements were deactivated (death) by multiplying their stiffness by 1.0 × 10 E6 to assume material failure and complete the iterative analysis. The element death analysis based on the obtained direct stiffness method was performed again to complete the iterative simulation. A total of 20 iterations were performed in this simulation. The high strain (>3000 μ) element located around the screw and occurring in each result from consecutive iterations can be displayed to trace out the micro-crack propagation pattern.

### 2.3. Mandibular Reconstruction Surgery and Radiation Treatment

In order to verify the element death analysis feasibility around the fixation screws for micro-crack prorogation, two skeletally mature New Zealand rabbits weighing around 4kg were used to perform the animal study. These two rabbit-specific reconstruction implants were designed according to the previous protocol and fabricated using a metal 3D printer (AM400, Renishaw, Gloucestershire, UK). One rabbit received radiation treatment and the other did not. The differences in bone remodeling caused by radiation treatment or not after surgery were compared. The animal experiment was reviewed and approved by the ethics review committee of the Institutional Animal Care and Use Committee (IACUC) of Master Laboratory CO., Ltd. (IACUC No.: MI20180801(1 January 2019–31 December 2019)).

**Figure 3:**
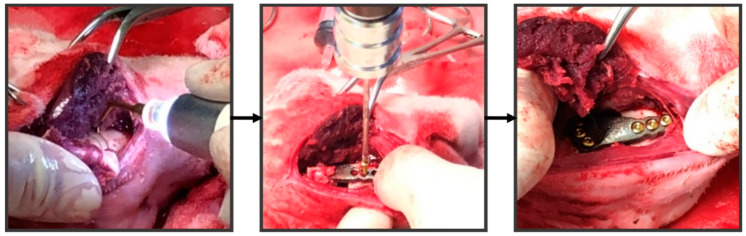
Animal experiments to place a specific reconstruction implant in the right corresponding region of the rabbit mandible.

Animal experiments were performed using intramuscular injection (IM) to mix ketamine (Imalgene 1000, Merial Laboratoire de Toulouse, Toulouse, France) 35–44 mg/kg BW and xylazine (Rompun, Bayer Korea Ltd., Ansan-si, Gyeonggi-Do, Korea) 5–10 mg/kg BW for surgical anesthesia. The rabbit was placed in a lateral position and the corresponding reconstruction position was cut using an ultrasonic bone saw (Via-Tech Biomedical Co., Ltd., Taichung, Taiwan) to place the specific reconstruction implant and fix it with 6 bone screws (Tandry, Microware Co., Ltd., Taichung, Taiwan) (Figure 3). Antibiotics (enrolloxacin 5 mg/kg, sid*7, SC) and analgesics (ketoprofen 2 mg/kg, sid*7, SC) were applied for 4 weeks. The rabbit health, food, and water intake and body weight were recorded after the surgery.

**Figure 4:**
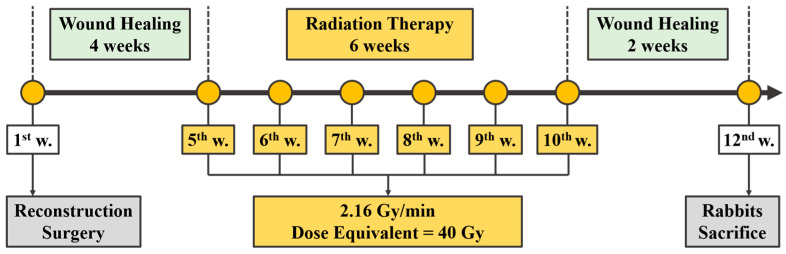
Time course of radiation treatment after reconstruction surgery.

For radiation treatment, one rabbit was placed on a disc-shaped radiation platform (Isotope Lab, National Tsing Hua University, Hsinchu City 300, Taiwan) 20 cm away from the central radiation source (60Co) to perform the treatment. The treatment protocol was scheduled once a week for 6 weeks and each dose was around 2.16 Gy/min, lasting for 3 min. The accumulated total dose was around 40 Gy after treatment [20]. Figure 4 shows the experimental program diagram after surgery. Two experimental rabbits were sacrificed 12 weeks after the surgery. The corresponding reconstruction implant with 6 screws and surrounding hard tissue were sectioned and placed in alcohol to dehydrate according to the 20%–40%–60%–80%–100% alcohol concentration sequence. The sample was then embedded, sliced, and ground to dye the sample with blue color for bone tissue and implant identification.

## 3. Results

**Figure 5:**
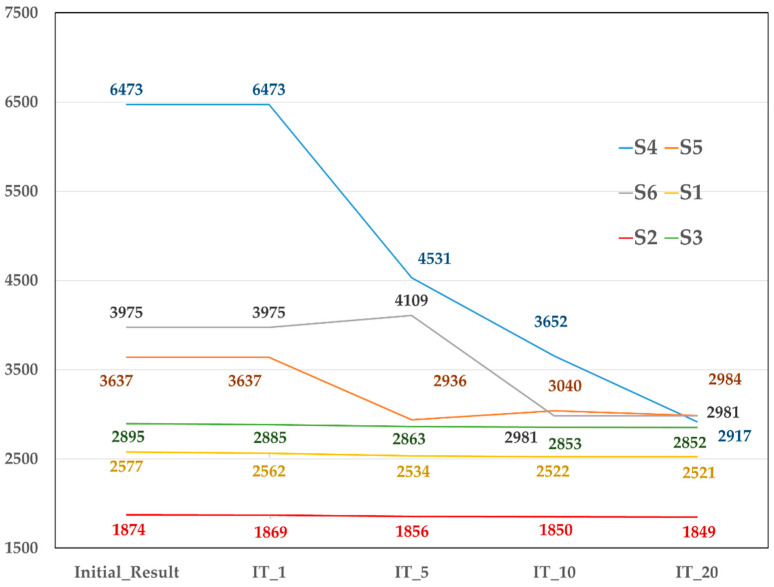
The amount of strain change gradually stabilized in S4, S5, and S6 screws with the increase in the number of iteration analyses.

The results showed that the strain values for the surrounding bone with the S5, S6, and S7 fixation screws were much larger than the corresponding positions for the S1, S2, and S3 screws. The strain values of the surrounding bone for the S4, S5, S6 fixation screws were greater than 3000 μ in the initial analysis. This implied that the bone entered the overload stage and started to deteriorate. With the increase in the number of iteration analyses, the amount of strain changed and gradually stabilized (Figure 5). However, the micro-movement began to gradually increase. The bone strain around the S4, S5, and S6 fixation screws was less than 3000 μ, while the micro-movement of the S4, S5, and S6 screws increased by 0.73%, 0.34%, 0.19% (Table 2). The screws were also beginning to loosen.

**Figure 6:**
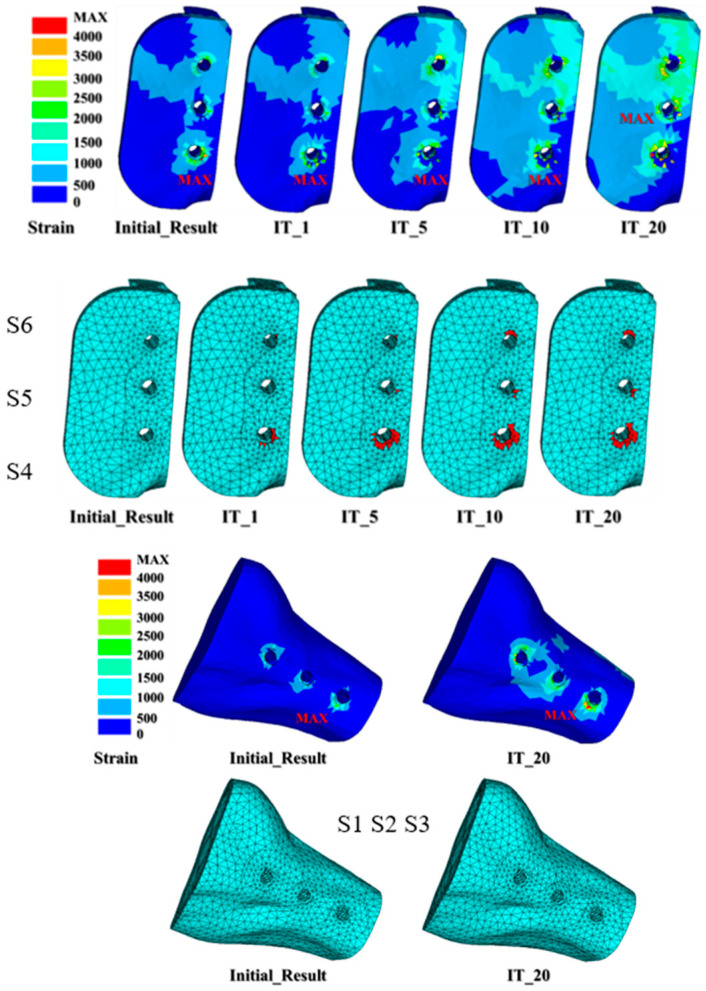
The micro-crack propagation trend, i.e., strain value > 3000 μ at the element around the screw can be represented by the element death number and locations during the iteration analysis process.

**Figure 7:**
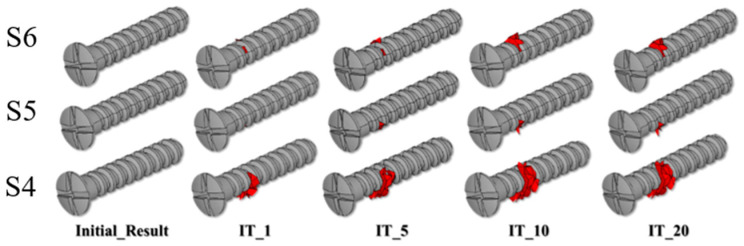
The element death trend around the screw can be represented by the number during the iteration analysis process.

The micro-crack prorogation trend can be represented by the number and element death locations during the iteration analysis process (Figure 6 and Figure 7). Table 3 presents the dead element number at the initial, first, five, ten, and 20 iterations. The initial crack occurred around the S4 fixation screw (IT_1) and 46 elements were dead. After performing five iterations (IT_5), the number of dead elements rose to 121 and the micro-cracks around the S4 screw expanded. The element around the S4 screw continued to deteriorate and 160 elements at the upper circumference of the screw began to deteriorate after ten iterations (IT_10). Most of the bone around the S4 screw was damaged and the upper circumference of the S6 screw also had cracks that continued to twenty iterations (IT_20) (Figure 6 and Figure 7). However, there was no damage around the S1, S2, and S3 fixation screws.

**Figure 8:**
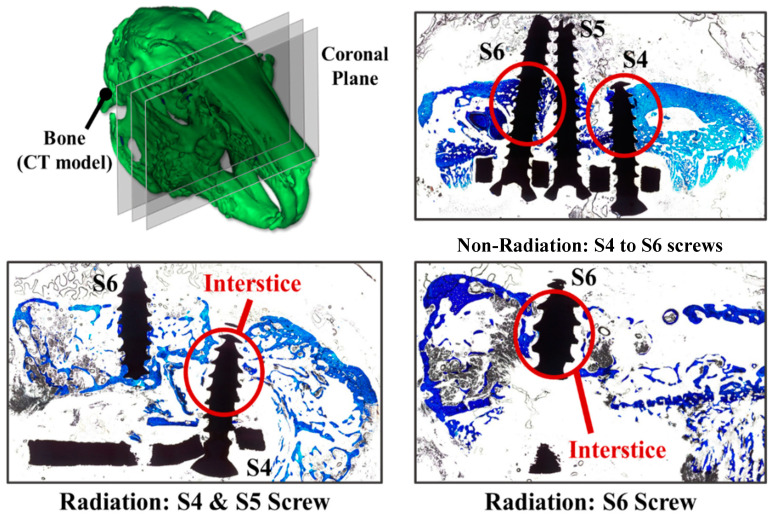
The hard tissue section strain results. Red circle: bone remodeling region around the screws.

The hard tissue section strain results are shown in Figure 8. The blue area is the stained bone and the white area is the unstained area which represents the bone loss area. Obvious micro-gaps (white color) exist around the S4 and S6 bone screws which imply that the bone loss (damage) is relatively high under the radiation treatment. In the control group without radiation treatment, the bone around the S4 and S6 bone screws was in close contact with the screw threads (blue color) and there were no significant gaps.

## 4. Discussion

Poort et al. placed dental implants into a mandible using mini-pegs and performed radiation treatment with different radiation doses for 26 weeks. The results showed that higher doses would reduce the bone remodeling rate and make the wound unable to heal [21]. Screw loosening after surgery can therefore be avoided through modified reconstruction implant/fixation screw design if the bone screw loosening caused by radiotherapy can be predicted or diagnosed in advance after the mandibular reconstruction surgery. 

In the mechanical biological theory, bone damage caused by 3H theory to bone cells means that the bone resorption threshold caused by normal bone overload is reduced. The threshold value and the related bone remodeling theory have been quantified with mechanical stimulation stress, strain, and energy. Many scholars integrated FE analysis and bone remodeling theory to simulate the long-term impact of changes in mechanical factors on bone after total hip replacement or dental implant placement [12,13,14,15,16,17]. However, the radiation treatment influences had not been taken into consideration for bone remodeling after surgery until now.

Element birth and death technique are often used in physical phenomena where the model wants to add or remove materials, such as material welding, crack accumulation, or material rupture and other behavior simulations. The definition of “element death” is not to remove elements but to make the element’s stiffness multiply 1.0E-6 (i.e., the rigidity approaches 0) and then perform subsequent analysis. The element birth does not involve actually adding specific elements to the model but constructing a complete model and hiding the birth elements and proliferating a specific element according to the actual needs in the analysis process [22].

This study used the element death technique provided in advanced FE analysis combined with bone remodeling theory to simulate the micro-crack propagation around a fixation screw using iteration calculation. Many studies have used quantifiable bone remodeling mechanisms and mathematical equations to take the external stimulus strain as an indicator [15,16,17]. The overload strain value threshold was modified from 4000 μ in a balanced situation to 3000 μ induced by radiotherapy. The bone remodeling mechanism was destroyed and the lazy zone repair area became narrow. This phenomenon indicated that the maximum bone load capacity to withstand an external load was decreased and bone remodeling entered the overload status when the bone was subjected to more than 3000 μ strain [23]. Subsequent iteration calculations can simulate fixation screw loosening caused by the bone loss around the screw at different time stages after the reconstruction surgery.

The simulated results show that the area with a strain exceeding 3000 μ initially occurs below the proximal end of the S4 bone screw. This means that the bone in this area is damaged and absorbed due to excessive load. With the calculation of iterations, the bone damage area range gradually expanded until a state of convergence was established, reaching 20 iterations. Recording the elements’ pattern of death during the iteration calculation can simulate the bone damage micro-crack propagation. The dead elements also represent repair ability loss and induce micro-movement in the bone screw until loosening. The stress is then transmitted into the rest of the bone screws and corresponding surrounding bone under continuous iteration calculations. Among the S4, S5, and S6 screws, the S6 bone screw bears the largest load after bone loosening around the S4 screw and becomes damaged. This causes the bone around the S6 screw to produce high strain, causing further bone damage. Therefore, it is possible to understand how bones are affected by radiation treatment after surgery through micro-crack propagation simulations and the bone screw micro-movement results. 

The total radiation treatment dose for rabbits in this study was set to 60 Gy and completed in 6 weeks. The hard tissue slice results showed that the rabbit mandible bones after radiation treatment are indeed relatively loose and this was consistent with the 3H theory in the aforementioned background. Micro-gaps were found around screws S4 and S6 after radiation treatment in in vivo experimental rabbits. This once again proved that the reconstruction implant load plus the radiation effects would change the bone remodeling mechanism. This mechanism interferes with bone repair, causing the possibility for osteoradionecrosis.

Although iterative analysis combining element death technology and bone remodeling theory in this research was verified with in vivo animal experiments, there are still some limitations on the parameters used in our simulations. These limitations include 1. oblique occlusal force applied on the incisors as the load condition in all simulations due to complicated occlusal behavior may cause difficulties to numerical convergence [18,19] and 2. the over load strain value threshold was assumed to be 3000 μ because no literature clearly indicated its value. 

## 5. Conclusions

This study concluded that the FE death technique combined with bone remodeling theory can simulate the bone loss mechanism around fixation screws affected by radiation treatment after surgery. The results showed that the FE simulation developed in this study can provide a better predictive diagnosis method for understanding fixation screw loosening mechanism.

## Figures and Tables

**Table 1:** Elastic modulus and Poisson’s ratios used in this study.

Material	Young’s Modulus	Poisson’s Ratio
Cortical Bone	13,700 MPa	0.3
Cancellous Bone	1370 MPa	0.3
Implants (Ti-6Al-4V)	110,000 MPa	0.35
Bone Screw (Ti-6Al-4V)	110,000 MPa	0.35

**Table 2:** Micro-movement of the S5, S6, and S7 screws at different iterations (unit: mm).

	S4_Dis.	S5_Dis.	S6_Dis.	S4_%	S5_%	S6_%
Initial results	0.000683	0.000596	0.000522			
IT_1	0.000685	0.000597	0.000522	0.29	0.17	0.00
IT_5	0.000687	0.000598	0.000523	0.59	0.34	0.19
IT_10	0.000688	0.000598	0.000523	0.73	0.34	0.19
IT_20	0.000688	0.000598	0.000523	0.73	0.34	0.19

IT: iteration of calculation.

**Table 3:** Dead element number at the initial, first, five, ten, and 20 iterations for all screws.

	S1	S2	S3	S4	S5	S6
Initial results	0	0	0	0	0	0
IT_1	0	0	0	46	1	5
IT_5	0	0	0	121	6	10
IT_10	0	0	0	160	9	23
IT_20	0	0	0	164	10	23
